# UniProt data pruning and the growing invisibility of the dark proteome

**DOI:** 10.1093/nargab/lqag115

**Published:** 2026-10-15

**Authors:** Ali Al-Fatlawi, Michael Schroeder

**Affiliations:** Biotechnology Center, Center for Molecular and Cellular Bioengineering, Technische Universität Dresden, 01307 Dresden, Germany; Department of Surgery, Universitätsklinikum Erlangen, 91054 Erlangen, Germany; Information Technology Research and Development Center, University of Kufa, 54002 Najaf, Iraq; Biotechnology Center, Center for Molecular and Cellular Bioengineering, Technische Universität Dresden, 01307 Dresden, Germany

## Abstract

The protein resource UniProtKB has undergone a major, multi-stage reorganization of its protein space, implemented from release 2025_04 through release 2026_02. Analysis of UniProtKB and UniParc records accessed on 20 January 2026 showed that 125.9 million entries were last present in UniProtKB in 2025 and are now represented in UniParc, while ~200.0 million entries remained in UniProtKB at the access date. Of these, 58.9 million were designated for removal in release 2026_02, whereas 141.2 million were classified as retained. The reorganization retains all reviewed UniProtKB/Swiss-Prot entries (the expert-curated subset of UniProtKB), along with proteins from selected reference proteomes and unreviewed entries with additional biologically important data, including experimentally determined three-dimensional structures. The expected benefits include reduced redundancy, easier navigation, and a protein set that is more tractable for annotation and routine searches. However, the reorganization may reduce the visibility of protein sequences from uncultured microorganisms and metagenomic surveys, phylogenetically underrepresented lineages, particularly many archaeal groups, proteins belonging to the so-called “dark proteome,” and strain-level variants of well-studied species such as *Escherichia coli*. We argue that the main consequence is not the loss of the sequences themselves, because affected proteins remain recoverable through UniParc and related resources, but a reduction in their discoverability, annotation context, and accessibility within routine workflows. These effects are likely to be most important for biodiversity-oriented analyses, retrospective reuse of older datasets, studies of specialized protein families, and downstream pipelines that rely on UniProtKB as an annotation hub. As UniProt shifts toward a more explicitly reference-proteome-centered model, a critical question arises: What are the implications of UniProtKB contraction for the representation of evolutionary diversity across the tree of life, particularly in under-sampled and uncultured lineages?

## Introduction

UniProtKB, the UniProt Knowledgebase, is the central protein sequence and functional annotation component of UniProt. It comprises (i) Swiss-Prot, the manually reviewed section that was established to provide reliable protein sequences with rich manual annotation, minimal redundancy, standardized nomenclature, and extensive cross-referencing, and (ii) TrEMBL, the computationally annotated unreviewed section, which later extended coverage as nucleotide and genome sequencing accelerated. The historical roots of this structure go back to earlier protein sequence collections, especially Margaret Dayhoff’s Atlas of Protein Sequence and Structure, first published in 1965; the PIR Protein Sequence Database, which Amos Bairoch received as a computer-tape version in 1984 after it had evolved from the Atlas tradition; and Bairoch’s intermediate PIR+ work, developed in the mid-1980s to convert and enrich PIR-style protein sequence records before SWISS-PROT was released on 21 July 1986 [1]. UniProtKB therefore carries forward a layered legacy of protein sequence curation, annotation, nomenclature, and cross-referencing, while also serving as a contemporary protein annotation resource [2]. UniParc, the UniProt Archive, is a complementary sequence archive that stores protein sequences and preserves links to their source database records, including records that may no longer be present in the current UniProtKB view. This legacy matters for reproducibility because older datasets, publications, and experimental workflows may cite UniProt, Swiss-Prot, TrEMBL, PIR, or earlier protein-sequence accessions from specific historical releases. Although UniProtKB accession numbers are intended to provide stable identifiers, some records may no longer appear in the current UniProtKB view and may instead need to be traced through UniParc, entry history, or accession mapping. Consequently, release-aware accession mapping is an important component of reproducible downstream protein annotation analysis.

### Balancing coverage and quality

UniProtKB’s multi-stage reorganization was implemented across releases 2025_04 to 2026_02. Analysis of UniProtKB and UniParc records accessed on 20 January 2026 showed that 125 910 734 entries had last appeared in UniProtKB in 2025 and were represented in UniParc. Of the 199 995 428 entries present in UniProtKB on the access date, 58 846 526 were designated for removal, and 141 148 902 were classified as retained (Fig. 1 and Table 1). The reorganization was intended to reduce redundancy, improve search efficiency, and focus the default UniProtKB view on reviewed entries, selected reference proteomes, and entries with prioritized biological evidence. The taxonomic effects differed depending on whether absolute numbers or proportional retention were considered. Bacterial proteins dominated the affected records numerically, particularly among entries last present in UniProtKB in 2025. In proportional terms, however, Viruses and Archaea showed the strongest contraction, with only 12.5% and 22.6% of their respective entries classified as retained, compared with 32.2% for Bacteria and 62.5% for Eukaryotes. Exact counts are provided in Table 1. Protein-length distributions broadly overlapped between retained and removal-designated entries, particularly in Bacteria and Archaea, with moderate differences in Eukaryotes and more pronounced differences in Viruses (Fig. 2). Short proteins occurred among both retained and removal-designated entries, and direct inspection of the underlying length data identified no proteins of one, two, or three amino acids in either category. These results provide no evidence that removal was driven primarily by a uniform minimum-length threshold or by the preferential exclusion of putative sequence fragments.

**Figure 1.**
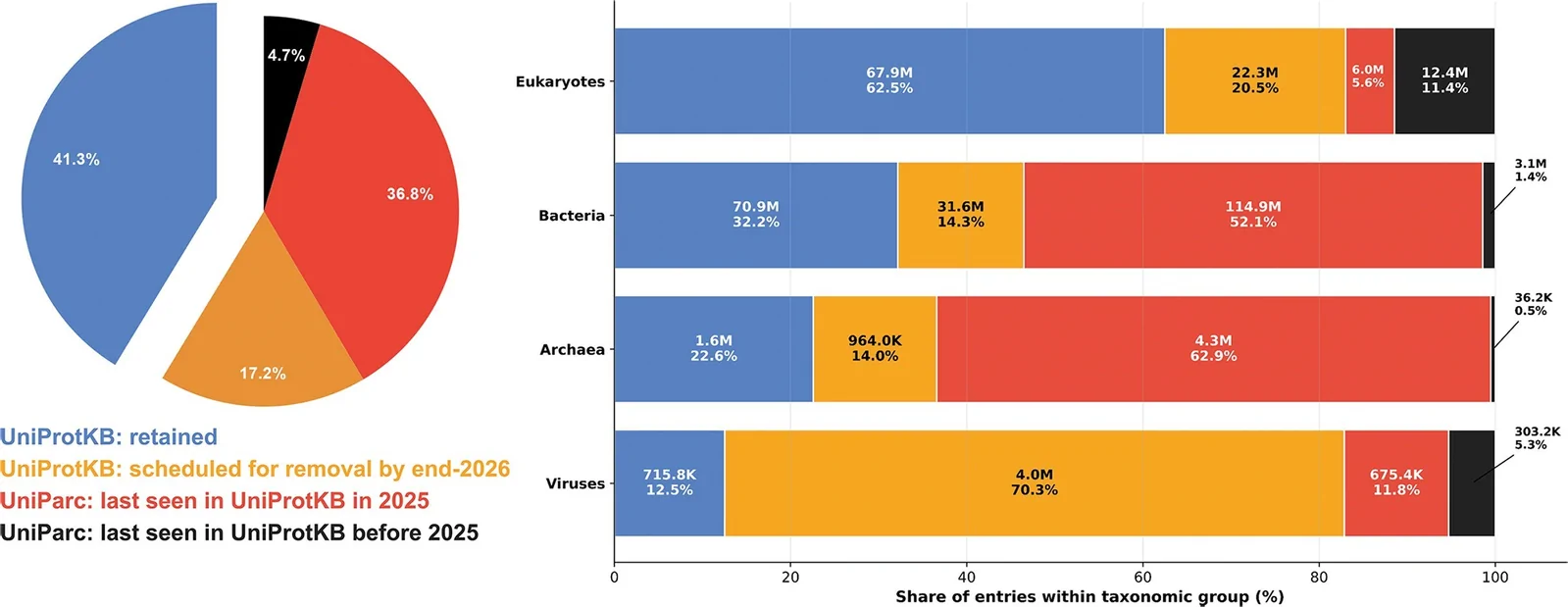
UniProtKB retention, designated for removal, and UniParc representation across taxonomic groups. The left panel shows the overall distribution of UniProtKB-associated protein entries across four status categories. Blue denotes entries retained in UniProtKB, including reviewed UniProtKB/Swiss-Prot entries, proteins from selected reference proteomes, and unreviewed entries with additional biologically important data. Orange denotes entries present in UniProtKB at the access date but designated for removal by the end of 2026. Red denotes UniParc entries whose last appearance in UniProtKB was in 2025, and black denotes UniParc entries whose last appearance in UniProtKB was before 2025. The right panel shows the proportional composition of these categories within Viruses, Archaea, Bacteria, and Eukaryotes; labels report the corresponding entry counts and within-group percentages. Bacterial entries dominate the affected records in absolute numbers, whereas Viruses and Archaea show the strongest proportional contraction, with 12.5% and 22.6% of entries retained, respectively, compared with 32.2% for Bacteria and 62.5% for Eukaryotes. Exact counts are provided in Table 1. Data access date: 20 January 2026.

**Figure 2.**
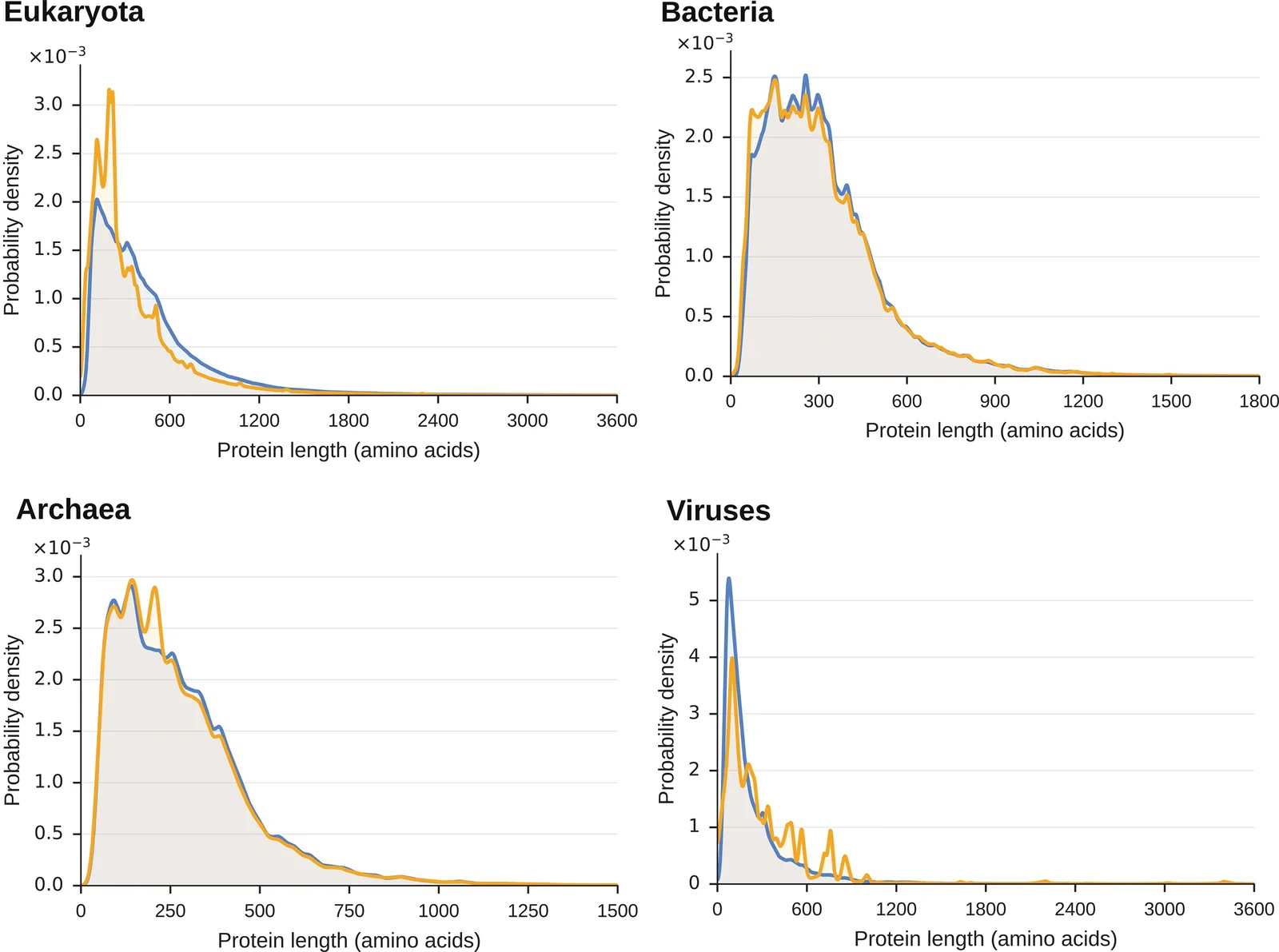
Protein-length distributions of retained and removal-designated UniProtKB entries across major taxonomic groups. For Eukaryotes, Bacteria, Archaea, and Viruses, it shows probability-density distributions across the full protein-length range. The distributions broadly overlap. No entries of one, two, or three amino acids were observed in either category.

**Table 1.** Exact numbers of UniProtKB-associated protein entries by taxonomic group and retention or archival status

	UniParc: last seen in UniProtKB before 2025	UniParc: last seen in UniProtKB in 2025	UniProtKB: designated for removal by the end of 2026	UniProtKB: retained
Viruses	303 166	675 445	4 018 054	715 792
Archaea	36 173	4 325 735	964 007	1 552 757
Bacteria	3 141 391	114 860 618	31 573 237	70 946 903
Eukaryotes	12 429 614	6 048 936	22 291 228	67 933 450
Total	15 910 344	125 910 734	58 846 526	141 148 902

Counts are shown for entries represented in UniParc whose last appearance in UniProtKB occurred before 2025 or during 2025, entries present in UniProtKB on 20 January 2026 but designated for removal by the end of 2026, and entries classified as retained in UniProtKB.

The substantially higher retention observed for Eukaryotes may instead reflect a combination of proteome selection, annotation density, multidomain architecture, and the availability of prioritized biological evidence. However, the present analysis does not isolate the relative contribution of these factors, and the taxonomic imbalance should therefore not be interpreted solely as a difference in biological redundancy. The stated rationale for UniProt’s reorganization is to reduce redundancy, improve search efficiency, and prioritize well-supported protein records. By reducing the number of entries from non-reference proteomes, including many strain-level and taxonomically unresolved records, UniProtKB can reduce redundancy and ambiguity in downstream annotation. This strategy shares the emphasis on high-quality representative genomes found in broader biodiversity initiatives such as the Earth BioGenome Project [3]. However, broad sequence coverage also introduces substantial redundancy, and large numbers of closely related entries can clutter similarity searches, increase computational costs, and complicate result interpretation [4].

A further consequence of pruning is a shift in how biodiversity is represented. Here, “representation of biodiversity” refers specifically to taxonomic evenness across species rather than to the total depth of sequence diversity within lineages. Reducing excessive strain-level representation can limit the dominance of intensively sequenced organisms and increase the relative visibility of selected species-level proteomes. As additional high-quality genomes become available, UniProtKB may expand in a controlled manner by incorporating proteins from newly represented species rather than repeatedly adding closely related sequence records. This shift from accumulation toward representative curation is intended to increase the proportion of well-supported entries in the default UniProtKB view. However, it also reduces sequence-level diversity within many lineages and raises concerns about long-term knowledge retention and the discoverability of strain-specific or lineage-restricted proteins. A release-wide comparison of redundancy at 100% and 90% sequence identity, together with a systematic analysis of the most highly repeated individual sequences, was beyond the scope of the present study but would provide a useful sequence-level complement to the retention analysis presented here.

### Consequences of UniProtKB pruning for the “dark proteome”

This curation comes at a price: the large-scale pruning of tens of millions of proteins risks obscuring vast, uncharacterized diversity. Global metagenomic surveys underscore the extent of poorly characterized protein space. For example, an analysis of 26 931 metagenomes and metatranscriptomes identified 1.17 billion protein sequences longer than 35 amino acids with no detectable similarity to sequences from 102 491 reference genomes or to Pfam entries. These sequences were grouped into 106 198 novel sequence clusters containing >100 members, revealing a vast unexplored component of microbial “functional dark matter” [5]. This result should not be read simply as evidence that UniProtKB lacks the relevant coverage, because the study benchmarked novelty against reference genomes and Pfam rather than UniProtKB itself, and because UniProtKB is a curated knowledge base rather than an exhaustive archive of all metagenomic protein fragments. Instead, it exposes a broader trade-off: curation improves reliability, redundancy control, and functional interpretability, but streamlined reference resources can make weakly characterized, lineage-restricted, environmentally derived, or uncultured-organism protein diversity harder to recover, compare, and interpret in downstream analyses. The central issue is therefore not only sequence absence but also reduced contextual discoverability: rare sequence neighborhoods, remote homology relationships, and release-specific accession histories may become difficult to track when low-confidence or poorly annotated records are removed from the primary knowledge base view. Coverage of uncultured organisms remains particularly constrained when available evidence derives mainly from metagenome-assembled genomes rather than isolated genomes. This reflects a quality-control trade-off rather than simple neglect: UniProt’s workflow prioritizes high-quality proteomes from species with recognized taxonomy and formal scientific names, while assemblies with unresolved quality, redundancy, or taxonomic issues may be less likely to be selected for reference-proteome status.

UniProt’s retention policy also preserves well-characterized proteins, including all entries with experimentally determined three-dimensional structures. The concern remains for “dark” proteins lacking structural information or recognizable homology, some of which may no longer appear in default UniProtKB searches and may instead need to be recovered through UniParc or other resources. Many affected orphan sequences may therefore become accessible primarily through UniParc rather than through standard UniProtKB searches. As a result, some proteins from extremophiles, uncultured microbes, or rare eukaryotic lineages (precisely those needed for evolutionary and ecological insights) may be removed from the main UniProtKB search index and represented primarily in UniParc. Affected sequences remain stored in UniParc and can be retrieved via the Proteomes portal but no longer appear in standard UniProtKB results [6]. These affected sequences are not synonymous with the dark proteome as a whole, because many removed entries may have recognizable homologs or existing annotations. Nevertheless, the affected set may include proteins belonging to the “dark proteome,” broadly defined as proteins lacking structural information or clear homology and therefore difficult to interpret functionally [7].

This issue is not only about individual orphan proteins; it becomes more consequential when the pruned sequences are unevenly distributed across the tree of life, because losses concentrated in uncultured or deeply branching lineages can reshape which parts of biodiversity remain visible in routine UniProtKB-based analyses. The impact on biodiversity studies is significant. Nearly all deep branches of life have been enriched by environmental sequencing. For example, recent work on terrestrial hot springs recovered 2949 archaeal genomes spanning 12 phyla and yielded 575 novel taxa (~19% of MAGs) [8]. However, this comes with the trade-off that within-species and strain-level redundancy is drastically reduced. The impact of this trade-off is particularly visible in Archaea. In the dataset analyzed here, only 22.6% of archaeal entries were classified as retained, while 77.4% were already represented only in UniParc or were designated for removal (Fig. 1 and Table 1). Consistent with this, our organism-level analysis indicated that many deeply branching archaeal taxa (particularly uncultured and Candidatus lineages known primarily from metagenomic assemblies) lose nearly all of their protein entries, in some cases retaining none in the curated UniProtKB. As a consequence of this pruning strategy, some evolutionary lineages that lack cultivated representatives may become absent or strongly underrepresented in the default UniProtKB view, even though their sequences remain archived. Many of these taxa correspond to candidate or poorly resolved archaeal lineages whose functional and evolutionary properties remain largely unexplored. When entire clades are underrepresented, analyses risk becoming incomplete or systematically biased. For example, phylogenetic studies comparing protein families across Bacteria and Archaea rely on dense sampling to resolve deep evolutionary relationships. With only 22.6% of archaeal entries retained in the analyzed dataset, some branches may be under-sampled in inferred trees, potentially obscuring ancient duplications, lineage-specific innovations, or the correct rooting of the tree of life. This limitation can be partly addressed by combining UniProtKB with UniParc, metagenomic protein collections, and primary genome resources. However, these resources do not provide the same level of integrated annotation and quality control as UniProtKB.

The same trade-off is not confined to deeply branching archaeal lineages; it also appears at broader taxonomic scales, where pruning affects bacteria more strongly than eukaryotes and, within individual species, shifts the balance from comprehensive strain-level diversity toward selected representative proteomes. UniProt’s curation removes a substantial fraction of bacterial sequences (over 60%), whereas the proportion removed for eukaryotic sequences is considerably lower (below 40%). At the species level, filtering out “redundant” proteomes dramatically compresses sequence diversity even for well-studied organisms. *Escherichia coli* illustrates the tension between representative coverage and within-species diversity, but the situation is more nuanced than a one-reference-proteome model. UniProt’s public documentation states that: “*For each species, we generally use an automatic pipeline to select one proteome as the Reference Proteome - the proteome that we believe is the best representative of the proteins encoded by that species (for example, the Reference Proteome for Drosophila simulans is UP000000304 as it provides the best coverage of the protein space for the species). In addition, proteomes of well-studied model organisms and other proteomes of interest for biomedical and biotechnological research may be selected as Reference Proteomes by UniProt curators*” [6]. Fortunately, this policy is not currently applied as a strict one-proteome-per-species rule: UniProt retains multiple reference proteomes for some highly diverse or intensively studied species. As of 27 May 2026, UniProt listed nine publicly available reference proteomes for *E. coli*, as well as pan-proteome resources for selected taxa. The limitation is therefore not the complete absence of strain-level support, but that the main UniProtKB search experience increasingly privileges a curated subset over the full species pan-proteome. For questions centered on accessory genes, pathovar-associated proteins, or strain-specific adaptations, researchers may need to combine UniProtKB with pan-proteome files, UniParc, or primary genome resources.

In the analyzed dataset, multicellular organisms also showed substantial losses, with tens of thousands of proteins affected in species such as the pig (*Sus scrofa*) and the common carp (*Cyprinus carpio*). Likewise, uncultured bacteria, minor protists, and other lesser-known lineages face the same fate. Even as projects like the Earth BioGenome Project aim to sequence all ∼1.8 million named eukaryotes [3], thousands of proteins from understudied taxa were designated for removal from the main UniProtKB view and representation in UniParc, where they remain accessible but are no longer searchable by default. Any evolutionary analysis that samples sequences broadly across the tree of life thus risks bias: when analyses rely only on a restricted set of representative proteomes, deep relationships become harder to resolve, and rare “bridging” homologs may be lost. Some affected sequences originate from organisms adapted to extreme or unusual environments (deep-sea vents, hypersaline lakes, acidic springs, symbiotic niches, etc.). For researchers studying intraspecies variation, minor isoforms, or strain-specific adaptations, UniProtKB may no longer provide the same breadth it once did. They may need to rely on archival resources or primary genome data to find those sequences. While the sequences themselves remain stored in archives, locating and utilizing them now requires additional technical effort.

### Impact on functional annotation

A key question raised by UniProtKB pruning is how it affects the UniProtKB-visible representation of functional annotation resources such as InterPro and the associated identification of protein domains, families, and motifs. Many gene-function pipelines depend on similarity to previously characterized protein families. Proteins without matches to established resources such as COG or Pfam are often more difficult to include in comparative functional analyses and may remain uncharacterized. This can reinforce an annotation imbalance in which well-studied protein families accumulate further functional context, whereas poorly characterized proteins remain difficult to classify.

InterPro [9], for example, integrates signatures from multiple member databases to classify proteins and predict domains, active sites, binding sites, conserved sites, repeats, post-translational modification sites, families, and homologous superfamilies. InterPro annotations were not used to determine whether UniProtKB entries were retained or designated for removal. Rather, we applied the independently obtained UniProtKB status classifications to the InterPro-to-UniProtKB mappings to assess how pruning changes the UniProtKB-visible membership of protein families and domains. To assess how UniProtKB pruning affects the UniProtKB-visible representation of InterPro-annotated protein space, we downloaded the InterPro-to-UniProtKB mapping data from the InterPro download portal on 20 January 2026. In total, 163 355 728 UniProtKB protein entries carried at least one InterPro annotation, distributed across 49 674 unique InterPro entries. These annotations comprised multiple InterPro signature types. Because a single protein can carry several InterPro annotations, the following values represent annotation assignments rather than mutually exclusive protein counts: Domain, 427 647 419 assignments; Family, 318 628 994; Homologous superfamily, 304 535 484; Repeat, 58 061 087; Conserved site, 28 328 601; Active site, 6 200 452; Binding site, 5 682 165; and PTM, 1 646 516. After applying the UniProtKB status classification available on 20 January 2026, 115 906 513 InterPro-annotated proteins were classified as retained, whereas 47 449 215, corresponding to ~30% of the InterPro-annotated UniProtKB set, were designated for removal from the main UniProtKB view in release 2026_02. Thus, the issue is not that InterPro signatures themselves disappear, but that the UniProtKB-visible membership of many InterPro entries changes substantially. The effect is unevenly distributed across InterPro entries. Of the 49 674 unique InterPro entries analyzed, 1351 had >90% of their UniProtKB-associated proteins designated for removal in the analyzed status mapping. In some extreme cases, the loss is complete. For example, in the InterPro-to-UniProtKB mapping downloaded on 20 January 2026, IPR022748 was associated with 686 proteins, all of which were designated for removal. A follow-up check on 5 August 2026 showed that 593 of these proteins were no longer present in the current UniProtKB view, whereas 93 remained accessible. However, these 93 entries were included in UniProt’s published to_remove list and therefore remained designated for removal. As of 5 August 2026, it remained unclear whether UniProt would ultimately remove these entries or revise their status and retain them. IPR022748 is ~90 amino acids in length, occurs N-terminal to PF00361, and all mapped members are annotated as NADH dehydrogenase subunit 2. This example is notable because PF00361-associated NADH dehydrogenase subunit 2 proteins also recur in the embedding-space outlier analysis, which will be discussed later in the embedding space section of this paper, linking the InterPro-level loss to a specific removal-enriched region of the protein embedding landscape. When InterPro assignments are stratified by annotation type and taxonomy, the pruning effect is again uneven. These counts refer to annotation assignments rather than unique proteins because a single protein may carry several InterPro annotations. The largest absolute changes were observed in broad annotation layers: ~103.4 million Domain assignments, 97.5 million Family assignments, and 82.5 million Homologous superfamily assignments were associated with proteins designated for removal in the analyzed status mapping. However, the proportional impact is most extreme for viral annotations. Among viral proteins, ~97.3% of Active site assignments, 95.0% of Homologous superfamily assignments, 95.0% of Domain assignments, and 94.1% of Family assignments occurred in entries designated for removal in the analyzed status mapping. Archaeal annotations showed a more moderate but consistent contraction, with ~36%–41% of assignments associated with entries designated for removal across most InterPro types. Bacterial annotations were affected at roughly 30% across most categories, whereas eukaryotic annotations were generally less affected, although Family assignments showed a stronger contraction of ~28.3%.

Several viral InterPro entries are especially strongly affected. For example, IPR014817, corresponding to the HIV Gag protein, mapped to >63 000 proteins in our dataset, of which >99% were designated for removal. Similarly, IPR009032, the Vpu protein cytoplasmic domain superfamily, was associated with 76 122 viral proteins, and IPR006859, influenza B matrix protein 2, was associated with 11 251 viral proteins; in both cases, nearly all mapped members were designated for removal. From a curation perspective, such reductions may be beneficial because viral proteins often include many highly similar strain- or isolate-derived sequences, and reducing these records can decrease redundancy and improve routine search interpretability. However, the negative consequence is that the apparent prevalence and sequence diversity of these viral families in the default UniProtKB view are dramatically reduced. Researchers interested in viral evolution, strain diversity, host adaptation, immune escape, or retrospective comparison with earlier UniProtKB releases may therefore need to supplement the retained UniProtKB set with UniParc, InterPro mappings, or other archival sequence resources.

Overall, known domain and family signatures remain represented at the InterPro level, but their UniProtKB-visible protein membership can contract sharply. This distinction is important: pruning does not necessarily invalidate InterPro signatures, Pfam families, or profile-HMM models, but it changes which proteins users will encounter when they rely on the current UniProtKB view alone. For Pfam, hidden Markov models are built from curated SEED alignments, many of which are already composed largely or entirely of reference-proteome sequences. Therefore, the immediate concern is not wholesale loss of HMM performance, but reduced discoverability and contextual annotation for lineage-specific families whose representatives become less visible in the main UniProtKB interface. Researchers can partly offset UniProtKB contraction by supplementing UniProtKB with other large protein resources, especially for metagenomic or poorly represented taxa. The present analysis considers individual InterPro annotations and their protein membership rather than complete domain architectures. Whether pruning preferentially retains multidomain proteins or reduces the visibility of rare domain combinations remains an important question for future analysis. However, these resources are not turnkey substitutes: many differ in release cadence, annotation depth, versioning, taxonomic consistency, and long-term maintenance, which complicates their direct use as replacements for UniProtKB in reproducible workflows. The original InterPro/UniProt mapping files and the curated tables used for this analysis are provided through the repository described in the “Data availability” section.

### Pan-genome and core-genome

A pan-genome comprises the full gene repertoire observed across multiple strains of a species, including both core genes shared broadly and accessory genes restricted to particular lineages or ecological contexts. *Escherichia coli* is a classic example of an open pan-genome, in which newly sampled strains continue to expand the species-level repertoire. The relevant concern for UniProt is therefore not merely whether a species retains a reference proteome, but how much accessory, pathovar-associated, and strain-specific biology remains visible in default UniProtKB use. *Escherichia coli’s* total pan-genome exceeds ~13 000 genes, whereas a single reference strain typically contains ~4300 genes. Analyses limited to one representative proteome may therefore exclude thousands of accessory genes found in alternative strains [10]. This difference between the core and pan-genome is not limited to bacteria but is also observed in some eukaryotes, especially plants. In one study, the core genome of rice (*Oryza*) contained ~10 000 genes, whereas the pan-genome exceeded 100 000 genes [11]. A similar argument can be made for Archaea, where, e.g. the *halophile Halorubrum ezzemoulense* has ~8400 pan-genes and only ~2600 core genes [12]. These gene-level estimates illustrate the difference between representative genome coverage and the broader protein-coding repertoire available across strains or accessions.

### Embedding-space analysis reveals broad global overlap but local contraction

We embedded ~200 million UniProtKB protein sequences using ProtT5 [13] and projected the resulting high-dimensional protein-level embeddings onto PC1 and PC2 using principal component analysis, as described in the “Data sources and analytical workflow” section. These entries were present in UniProtKB on 20 January 2026 and were classified as either retained or designated for removal. The analysis should be interpreted as a two-dimensional view of the embedding space rather than a complete representation of its high-dimensional structure.

To visualize the complete dataset without point overplotting, Figure 3a and b show two-dimensional binned protein counts for retained and removal-designated proteins, respectively, using the same PC1–PC2 coordinate space.

**Figure 3.**
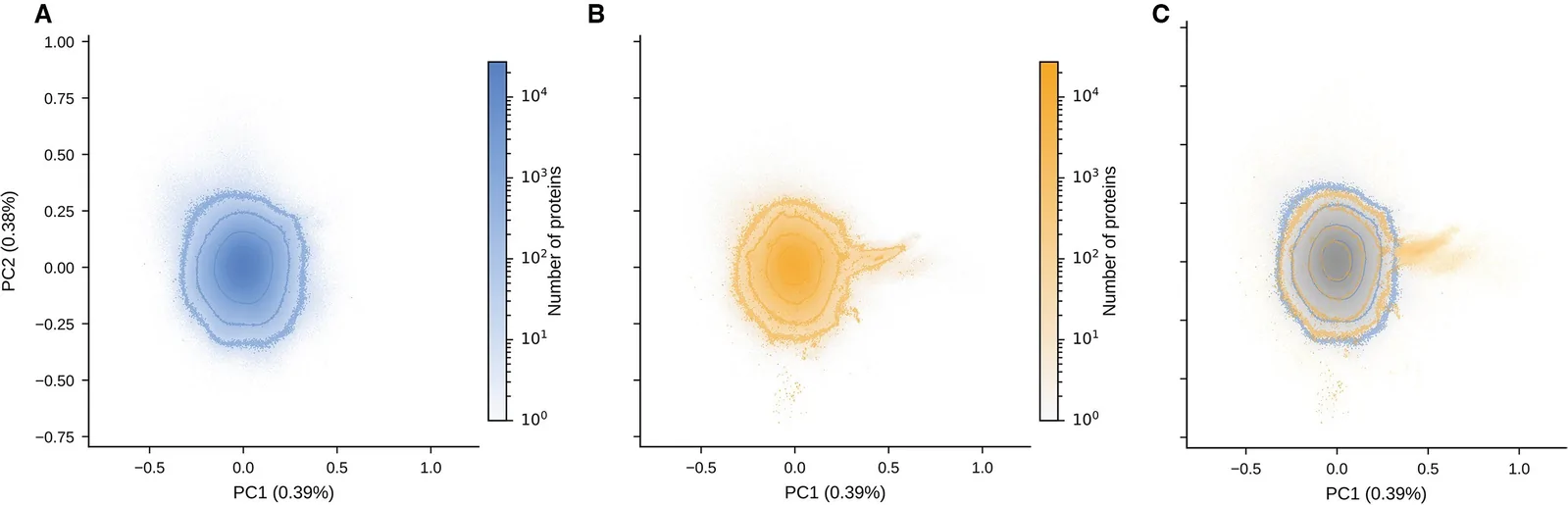
Distribution of retained and removal-designated proteins in the ProtT5 principal component analysis (PCA) space. Two-dimensional binned count distributions of (**a**) retained proteins and (**b**) removal-designated proteins. Color intensity represents the absolute number of proteins, while contours summarize the corresponding distributions. (**c**) Spatial distribution of peripheral removal-designated proteins in removal-enriched regions. The gray background represents all proteins, and the blue and orange contours show the complete distributions of retained and removal-designated proteins, respectively. The orange foreground represents removal-designated proteins located in regions containing at least 75% removal-designated proteins, exhibiting positive enrichment relative to the global removal frequency. All retained and removal-designated proteins were included in the analysis without sampling.

At the global scale, the retained and removal-designated distributions showed extensive overlap in the central region of the PC1–PC2 projection (Fig. 3a and b). Retained proteins covered the principal range occupied by the complete dataset, whereas the removal-designated distribution additionally extended into distinct peripheral regions, most prominently toward positive PC1 values. Thus, the retained set broadly covers the central structure visible in this projection, while local differences remain evident at its periphery.

To examine these local differences, removal-enriched regions were defined as spatial bins in which at least 75% of proteins were designated for removal. Figure 3c maps removal-designated proteins occurring in these regions against the distribution of all proteins, with blue and orange contours indicating the complete retained and removal-designated distributions, respectively.

Given the substantially lower global frequency of removal-designated proteins, the 75% criterion identifies regions with strong local enrichment rather than regions that merely reflect the overall class proportions. Notably, deep protein embeddings are known to capture structural and functional protein features [14], but localized enrichment in principal component analysis (PCA) space was treated as a hypothesis-generating signal and was therefore characterized directly rather than assigned a biological interpretation from the embedding coordinates alone. These removal-enriched peripheral regions were therefore examined directly. We analyzed proteins from the prominent right-hand peripheral region in Fig. 3c, using PC1 > 0.5 to define the outlier set. This set comprised ~41 000 proteins designated for removal and was strongly enriched for metazoan sequences, especially Chordata (32 208 proteins) and Arthropoda (8069 proteins). This region was dominated by a limited number of recurrent Pfam signatures, although ~4000 proteins lacked detectable Pfam assignments and were therefore treated separately as an unannotated subset. Among the Pfam-annotated proteins, the most frequent taxonomy-Pfam combinations were PF06444/PF00361 in Chordata (21 828 proteins) and Arthropoda (325 proteins), PF00895 in Chordata (8588) and Arthropoda (2001), PF00507 in Arthropoda (876) and Chordata (137), and PF02790 in Arthropoda (912).

Functional inspection of these dominant Pfam patterns indicated enrichment for small hydrophobic mitochondrial membrane proteins involved in oxidative phosphorylation. In particular, the co-occurrence of PF00361 and PF06444 was consistent with ND2-like, Complex I-associated proteins, whereas PF00895 was consistent with ATP8-like, Complex V-associated proteins. The Pfam-unassigned subset indicates that not all proteins in these peripheral regions can be functionally classified from domain annotation alone, but its presence does not remove the dominant signal from the annotated majority. Sequence-length analysis further argued against a simple fragment explanation: outlier proteins were concentrated between ~50 and 500 amino acids, with distinct density peaks at ~65–70, 170–180, and 340–350 amino acids rather than a single spike of extremely short sequences. We therefore interpret these peripheral removal-enriched regions as coherent neighborhoods enriched for mitochondrial membrane-protein classes, while noting that a minority of sequences remain unclassified by Pfam. Overall, retained proteins broadly cover the central structure visible in the PC1–PC2 projection, whereas specific peripheral neighborhoods are strongly enriched for removal-designated proteins, including regions enriched for metazoan mitochondrial oxidative phosphorylation proteins. The spatially non-uniform distribution therefore indicates localized contraction within this projection, with these protein classes, together with a smaller Pfam-unassigned component, becoming less visible in standard UniProtKB-centered analyses.

### Bioinformatics landscape: usability versus completeness

A leaner UniProtKB offers clear usability benefits. Relative to the combined protein space represented before and during the reorganization, the retained UniProtKB set is >50% smaller. Routine analyses are therefore expected to run faster and produce fewer redundant hits [4]. BLAST searches, profile-HMM scans, identifier mappings, local database mirrors, and release updates may therefore require fewer computational and storage resources. Maintaining local mirrors and updating databases also becomes easier as data volume drops. In short, a more streamlined knowledge base improves general workflows and user experience by focusing on high-quality reference proteomes. However, these gains come with a trade-off in completeness that researchers must address. Many bioinformatics pipelines (especially in genomics and proteomics) were built under the assumption of UniProtKB’s formerly comprehensive coverage. If a protein sequence is absent from the current UniProtKB view, evidence associated with that specific sequence may be missed in workflows that rely exclusively on UniProtKB. For example, mass spectrometry-based proteomics depends on matching spectra to a sequence database, and a specific protein sequence that is absent from the search database cannot be directly identified by a conventional sequence-database search [15]. Archival sequence retention alone does not fully preserve proteomics utility. Database searching and peptide interpretation often depend on annotated biological context, including signal peptides, propeptides, mature-chain boundaries, transmembrane segments, proteolytic processing, and soluble alternative products. This is particularly relevant for membrane proteins, for which soluble variants may arise through alternative splicing or proteolytic shedding; Möller *et al*. compiled and reported soluble variants and generated putative variant datasets to improve the interpretation of transcriptomic and mass-spectrometry-based proteomic data [16]. Such splicing and shedding events can alter the expected N- or C-terminal peptides, thereby affecting peptide detectability and protein identification.

UniParc can preserve sequence existence and accession continuity, but it does not substitute for the feature-rich annotation layer available in UniProtKB. This issue remains relevant to downstream annotation, cross-study comparison, and retrospective reanalysis in metaproteomics and microbiome research, where samples often contain diverse, strain-specific proteins. Missing reference sequences may lead to unassigned peptide spectra or even incorrect identifications if a pipeline mistakenly assigns a spectrum to the closest database match rather than the true (absent) protein [15]. To maintain sensitivity in such cases, researchers may need to supplement the retained UniProtKB set with sequences from archival, sample-specific, or primary genome resources. Importantly, all sequences removed from UniProtKB remain available via UniParc or other repositories, so scientists can still retrieve complete proteomes and proteins as needed. The bioinformatics ecosystem therefore faces a new balance: a more navigable core database is obtained at the cost of additional effort to recover auxiliary sequences and annotation context when comprehensive coverage is required.

In environmental metaproteomics, however, UniProtKB is generally not used as the primary search space for MS/MS spectrum matching. The prevailing strategy is to search against a sample-matched metagenome-derived protein database, whereas large general resources such as NCBI-nr are often used iteratively or downstream for taxonomic and functional annotation. Our concern is therefore not that UniProtKB alone underpins all metaproteomics identifications, but that changes in UniProt visibility can still affect downstream annotation, cross-study comparability, and retrospective reanalysis workflows.

### Clinical observability of disease-specific protein forms

A related concern involves disease- or context-specific protein forms whose sequences and annotations may support clinical, proteomic, and translational research. An informative example of the observability issue raised here is UniProtKB entry A8E194, which is annotated as a human BCR-ABL1 fusion protein and was designated by UniProt for removal from UniProtKB/TrEMBL because it is not part of a reference proteome. The biological and clinical relevance of the BCR-ABL1 fusion itself is well established, including its role in Philadelphia chromosome-positive leukemia and its centrality as a therapeutic target [17, 18]. In addition, A8E194 has itself been used as a UniProt-derived BCR-ABL1 fusion-protein record in downstream computational work [19]. This example does not establish that clinically relevant fusion proteins are broadly or systematically removed. The present analysis did not quantify all entries carrying disease-related keywords, disease-variant annotations, or other indicators of clinical relevance, and the frequency of comparable cases therefore remains unknown. Rather, A8E194 illustrates a narrower point: for disease-associated fusion products, discoverability may depend on whether users search archived records, historical entries, or external disease-specific resources in addition to standard UniProtKB workflows.

### Implications for analysis pipelines and AI

Modern protein analysis pipelines, including structure-prediction and protein-language-model workflows, rely on large sequence collections during model training, database retrieval, multiple-sequence-alignment construction, or downstream annotation. If a model were trained exclusively on the retained UniProtKB set, many rare-lineage sequences designated for removal would be excluded, potentially limiting its ability to generalize to underrepresented protein families and lineages. In practice, however, leading methods already integrate external sequence reservoirs. For example, AlphaFold2’s pipeline [20] constructs multiple sequence alignments using several large sequence resources, including UniRef90, MGnify [21], and the Big Fantastic Database (BFD) [20]. BFD was constructed from >2.4 billion input protein sequences and contains families covering ~2.2 billion sequences. Because the AlphaFold2 pipeline draws on multiple sequence resources, its dependence on UniProtKB alone is limited. However, the present analysis did not test whether UniProtKB contraction affects prediction performance. Other tools may be affected differently. Foldseek [22], for instance, compares protein structures against large structure databases, including collections of UniProtKB-derived structure predictions. A reduced UniProtKB-derived structure collection could increase reliance on complementary predicted-structure resources, including metagenomic datasets. The contraction of the UniProtKB-visible protein set shifts greater responsibility to data scientists and tool developers, who may need to incorporate metagenomic, strain-specific, and variant datasets rather than assuming that the retained UniProtKB set provides sufficient coverage for every application. Such considerations apply to protein language models used for functional annotation and homology inference. A training set drawn exclusively from the retained UniProtKB set would contain fewer sequences and provide less direct representation of some rare or underrepresented lineages, which could affect model generalization in those regions of protein space. Fortunately, many recent efforts have already expanded beyond the curated set. Meta’s ESM Metagenomic Atlas, for example, provides predicted structures for ~600 million metagenome-derived proteins [23]. Thus, AI-driven protein analysis need not be limited to the retained UniProtKB set when pipelines integrate additional large and diverse sequence collections. The principal implication is therefore a shift in responsibility: tool developers and users need to document and deliberately select the sequence resources used for training, alignment construction, retrieval, and annotation rather than assuming that UniProtKB alone provides sufficient breadth.

### Pathways forward

UniProt’s reorganization does not eliminate access to the affected sequences, but it underscores the need for complementary strategies to preserve their discoverability and annotation context. We propose several measures:

- Release-specific mapping and accession resolution. UniProt should provide machine-readable, release-specific mappings that identify whether an entry was retained, removed from UniProtKB, merged with another entry, or represented only in UniParc. Stable accession-resolution paths should allow users to move from historical UniProtKB identifiers to the corresponding UniParc records without losing sequence provenance or links to associated publications.
- Archive flagging and prioritization. Entries no longer visible in UniProtKB but represented in UniParc could be tagged with metadata indicating their provenance, taxonomic origin, potential novelty, or previous UniProtKB status. This would help users and curators rediscover interesting sequences when they resurface in new studies.
- Auxiliary biodiversity resources. Develop or extend complementary resources to represent proteins from metagenomes, less frequently sampled strains, and other sequence classes that are not prioritized for routine UniProtKB display. Existing microbial resources such as MGnify [21] and IMG could contribute to this ecosystem, whereas alternative isoforms may require organism- or disease-specific repositories. These complementary resources could preserve broader sequence coverage without expanding the routine UniProtKB search space, provided that stable cross-references and clearly documented provenance are maintained. For example, specialized microbial enzyme catalogs or pan-genome libraries could capture this functional diversity without requiring full manual curation.
- Configurable search tools. Homology-search and genome-annotation tools should allow users to optionally include archived sequences. Users could be offered a configurable comprehensive search mode that queries UniProtKB together with UniParc and other selected sequence archives. Clear result labeling should distinguish UniProtKB status, annotation provenance, review status, and archival origin, allowing researchers to interpret each match according to the evidence available. Existing sequence-search interfaces already demonstrate that users can select among alternative search sets, providing a practical basis for such configurable retrieval.
- AI-assisted annotation and retrieval. Computational methods could complement expert curation by helping to prioritize archived sequences and recover their biological context. Large protein language models and structure-based search methods may help recover remote homologous relationships that are difficult to detect by conventional sequence similarity alone [24, 25], while family-level annotation resources can provide additional domain and functional context. Such systems should be framed as aids to prioritization and rediscovery rather than as replacements for expert review, particularly for clinically relevant proteins. In this sense, UniProtKB contraction revisits a longstanding bioinformatics problem: how to preserve findability and annotation continuity across changing resources while using computational methods to support large-scale prioritization and contextual recovery.
- Scalable and federated infrastructure. Longer-term development could explore architectures that preserve UniProtKB as a curated core while enabling efficient, federated retrieval across UniParc, metagenomic repositories, pan-genome resources, and specialist databases. Such an approach could improve scalability without requiring every sequence and annotation layer to be maintained within a single routine search space.

## Conclusion

UniProt’s reorganization reflects a longstanding trade-off between breadth and curatorial tractability rather than a simple retreat from biological diversity. The principal risk is the reduced discoverability of rare, lineage-specific, legacy, and some clinically relevant protein records in standard UniProtKB workflows. This risk can be mitigated through transparent, release-specific mapping files, stable accession-resolution paths to UniParc, and targeted analyses of the protein classes that become less visible. Integrating metagenomic sequence repositories with AI-assisted annotation and retrieval tools could further preserve access to the long tail of protein biodiversity without compromising UniProtKB’s curatorial focus.

## Data Availability

The source files and curated analysis data underlying this study are available on Figshare at https://doi.org/10.6084/m9.figshare.32628309. These include the UniProtKB status and UniParc last-seen-year tables underlying Fig. 1 and Table 1, the InterPro-to-UniProtKB status and the taxonomy mapping tables used for the functional-annotation analysis, and the length of all proteins, which are visualized and compared in Fig. 2.

## Data sources and analytical workflow

UniProtKB and UniParc data accessed on 20 January 2026 were analyzed separately for Viruses, Archaea, Bacteria, and Eukaryotes. UniProtKB accessions were classified by matching them to the published retained, “to_remove,” and “*unclassified_removed*” lists. For Fig. 1 and Table 1, the “to_remove” and “unclassified_removed” categories were combined as entries designated for removal. Taxon-specific UniParc tables were analyzed using the “Last seen” field, which was converted to a calendar year to distinguish records last present in UniProtKB during 2025 from those last present before 2025. The resulting taxon-specific status and UniParc tables were exported for downstream analysis. For the functional-annotation analysis, UniProtKB-to-InterPro mappings were obtained from “Interpro_protein2ipr.dat.gz,” and InterPro entry types were added from “Interpro_entry.list.” The independently derived UniProtKB status classifications and taxonomic groups were then assigned to each protein-InterPro mapping. Counts and percentages were calculated by InterPro entry, annotation type, taxonomic group, and UniProtKB status.

Protein sequences were embedded using the ProtT5-XL-UniRef50 encoder (Rostlab/prot_t5_xl_uniref50) implemented with the Hugging Face Transformers library. Sequences were limited to the first 2500 amino acids before tokenization and converted to space-separated amino-acid tokens; tokenizer-side truncation was additionally enabled with a maximum input length of 2500 tokens. Embeddings were generated one protein at a time with the model in evaluation mode. Final-layer token representations were mean-pooled over all non-padding positions according to the attention mask, yielding one 1024-dimensional vector per protein. GPU inference, when available, used automatic mixed precision, after which embeddings were converted to float32 for storage. The resulting vectors were stored with their protein identifiers in HDF5 files and subsequently repacked into aligned matrices containing up to one million proteins per file.

A single global principal component analysis (PCA) model was fitted to all ~200 million embeddings, rather than to a sampled subset, using streaming covariance statistics to avoid loading the complete embedding matrix into memory. The embeddings were centered using the global feature mean but were not otherwise scaled or normalized. Covariance estimation and eigendecomposition were performed in float64, and the first 50 principal components were retained. All protein embeddings were subsequently projected into the same PCA space, with the resulting scores stored as float32; PC1 and PC2 were used for visualization.

For visualization, the complete observed PC1–PC2 coordinate space was divided into an 800 × 800 equal-width grid. Each eligible protein contributed once to the spatial bin containing its coordinates, separately for retained and removal-designated proteins. Color intensity represents the absolute number of proteins per bin on a logarithmic scale, and contours were derived from the same binned counts. All eligible proteins were included without sampling, kernel density estimation, coordinate clipping, or minimum-count filtering. Bins in which at least 75% of proteins were designated for removal were classified as removal-enriched. Empty bins were excluded from the calculation of the removal fraction, whereas non-empty bins were retained without a minimum-count threshold. For downstream characterization, the positive-PC1 peripheral region was defined as removal-designated proteins occurring in removal-enriched bins with PC1 > 0.5.
